# Supplementary material for: Detecting nuance in conspiracy discourse: Advancing methods in infodemiology and communication science with machine learning and qualitative content coding
Source: PLoS One. 2023 Dec 20;18(12):e0295414. doi: 10.1371/journal.pone.0295414 (PMC10732406; doi:10.1371/journal.pone.0295414)
Supplement: S1 Appendix — (PDF) [file pone.0295414.s002.pdf]

Step 1 *Collect data from API*

Objective: Collect relevant posts from API using keywords related to discourse

Twitter Public Streaming API

Filter criteria

Dates: March 25th – April 3rd, 2020

Keywords: “[5G]”
“[coronavirus]”
“[covid-19]”

Total Dataset

256,562 tweets resulting from date and keyword filters

Step 2 *Process data using topic modeling*

Objective: Use Biterm Topic Modeling (BTM) to cluster posts together based on textual similarity

Total Dataset

256,562 tweets

Topic Clusters

0 1 2 3
4 5 6 7
8 9 10 11
12 13 14 15
16 17 18 19

Note: Other topic models such as LDA or BERT can be used instead. See Methods section for how to determine number of topic clusters to select

Step 3 *Characterize topic clusters*

Objective: Content code the top 10 most shared posts associated with each topic cluster

0 1 2 3
4 5 6 7
8 9 10 11
12 13 14 15
16 17 18 19

Posts can be coded for containing misinformation or corrections, stance, and inductive coding themes. Characteristics of user accounts (ex – affiliations, suspended status) can also be coded during this phase

Tweet_ID	Tweet Text	Sentiment	num_retweets	topic	Inductive Themes	Misinformation Classification	Suspended	Deleted	Affiliation
'12476890	Hard Pass...consider that everyone who receiv	1	1132	4	Anti-vax	Conspiracy			
'12458488	If people knew what was unfolding & what sat	1	908	4	Elite	Conspiracy			Religious Leader
'12443236	5G Bill Signed Into Law While Everyone Is Distr	1	855	4	David Icke	Conspiracy	1		
'12450078	All those morons who considerd me a conspir	1	562	4		Conspiracy			
'12290436	What’s the common thread that goes through	1	547	4	Anti-vax	Conspiracy		1	
'12479258	I wrote about all the ways conspiracy theorists	-1	409	4		Misinformation Correction			Media
'12453261	What you need to know about Bill Gates and h	1	342	4	Elite	Conspiracy		1	
'12376770	@realDonaldTrump @WHO #event201 👀It’s	1	336	4		Conspiracy	1		
'12454669	The crazies are out again."Soon ALL will be coe	-1	270	4	pro vaccine	Misinformation Correction			Medical Affiliate
'12454538	Bill Gates, WHO chief, killer and psychopath.S	1	189	4	Elite	Conspiracy		1	

Step 4 *Identify relevant topic clusters*

Objective: Identify clusters for further investigation based on content coding

Calculate the average % of misinformation posts within a cluster. Also calculate average % for stance and inductive themes

Misinformation Corrections Mixed

0 1 2 3
4 5 6 7
8 9 10 11
12 13 14 15
16 17 18 19

0 1 3
4 5 6 7
8 9 10 11
13
16 17 18 19

Select clusters that contain relevant signal

Step 5 *Run sentiment analysis*

Objective: Calculate sentiment scores for all posts within the dataset

Use LIWC (or software of choice) to run sentiment analysis on entire dataset

Calculate the average sentiment for each topic cluster and identify clusters in 90th percentile

Topic Clusters by sentiment

0 10 17

Negative emotion

Death

Clusters with sentiment categories of interest (ex - Negative emotion, Death) can be further investigated
