# Supplementary material for: Detecting nuance in conspiracy discourse: Advancing methods in infodemiology and communication science with machine learning and qualitative content coding
Source: PLoS One. 2023 Dec 20;18(12):e0295414. doi: 10.1371/journal.pone.0295414 (PMC10732406; doi:10.1371/journal.pone.0295414)
Supplement: S1 Table — (PDF) [file pone.0295414.s003.pdf]

		ACCOUNT STATUS		AFFILIATION			
**TOPIC**	Unique users	Suspend	Deleted	MedSci	Media	Relig	Govt
**0**	3	**66.7%**	*0.0%*	*0.0%*	*0.0%*	*0.0%*	*0.0%*
**1**	9	**33.3%**	*0.0%*	**44.4%**	*0.0%*	*0.0%*	*0.0%*
**2**	7	*0.0%*	14.3%	*0.0%*	**71.4%**	*0.0%*	*0.0%*
**3**	8	**12.5%**	**37.5%**	**12.5%**	**12.5%**	*0.0%*	*0.0%*
**4**	7	**14.3%**	**14.3%**	**14.3%**	**14.3%**	*0.0%*	*0.0%*
**5**	9	**33.3%**	*0.0%*	*0.0%*	**11.1%**	*0.0%*	*0.0%*
**6**	7	**57.1%**	14.3%	*0.0%*	*0.0%*	*0.0%*	*0.0%*
**7**	6	**50.0%**	*0.0%*	**33.3%**	**16.7%**	*0.0%*	*0.0%*
**8**	8	**25.0%**	*0.0%*	*0.0%*	*0.0%*	*0.0%*	**12.5%**
**9**	7	**85.7%**	*0.0%*	*0.0%*	*0.0%*	*0.0%*	*0.0%*
**10**	10	*0.0%*	**40.0%**	*0.0%*	*0.0%*	*0.0%*	**10.0%**
**11**	9	**11.1%**	*0.0%*	**11.1%**	**11.1%**	*0.0%*	*0.0%*
**12**	6	**33.3%**	*0.0%*	*0.0%*	**16.7%**	*0.0%*	*0.0%*
**13**	9	**22.2%**	*0.0%*	**11.1%**	**44.4%**	*0.0%*	*0.0%*
**14**	10	*0.0%*	*0.0%*	**10.0%**	**30.0%**	*0.0%*	**30.0%**
**15**	8	**12.5%**	**12.5%**	**12.5%**	**12.5%**	**12.5%**	*0.0%*
**16**	3	**66.7%**	*0.0%*	*0.0%*	*0.0%*	*0.0%*	*0.0%*
**17**	9	**33.3%**	*0.0%*	**44.4%**	*0.0%*	*0.0%*	*0.0%*
**18**	7	*0.0%*	**14.3%**	*0.0%*	**71.4%**	*0.0%*	*0.0%*
**19**	8	**12.5%**	**37.5%**	**12.5%**	**12.5%**	*0.0%*	*0.0%*
